# Imaging-assisted anticancer nanotherapy

**DOI:** 10.7150/thno.38288

**Published:** 2020-01-01

**Authors:** Anshuman Dasgupta, Ilaria Biancacci, Fabian Kiessling, Twan Lammers

**Affiliations:** 1 Department of Nanomedicine and Theranostics, Institute for Experimental Molecular Imaging, RWTH Aachen University Clinic, Aachen, Germany.; 2 Department of Pharmaceutics, Utrecht University, Utrecht, the Netherlands.; 3 Department of Targeted Therapeutics, University of Twente, Enschede, the Netherlands.

**Keywords:** anticancer nanotherapy, cancer nanomedicines, nanotheranostics

## Abstract

Cancer nanomedicines are submicrometer-sized formulations designed to improve the biodistribution of anticancer drugs, resulting in less off-target localization, altered toxicity profiles, improved target site accumulation and enhanced efficacy. Together, these beneficial features have resulted in the regulatory approval of about a dozen nanomedicines for the treatment of solid and hematological malignancies. In recent years, significant progress has been made in combining nanomedicines with imaging, to better understand key aspects of the tumor-targeted drug delivery process, and to address the high inter- and intra-individual heterogeneity in the Enhanced Permeability and Retention (EPR) effect. Strategies explored in this regard have included the use of traditional imaging techniques, companion diagnostics and nanotheranostics. Preclinically, integrating imaging in nanomedicine and drug delivery research has enabled the non-invasive and quantitative assessment of nanocarrier biodistribution, target site accumulation and (triggered) drug release. Clinically, imaging has been emerging as a promising tool for patient stratification, which is urgently needed to improve the translation of cancer nanomedicines. We here summarize recent progress in imaging-assisted anticancer nanotherapy and we discuss future strategies to improve the performance of cancer nanomedicines in patients.

## 1. Introduction

Intravenously administered chemotherapeutics entail a number of drawbacks, such as poor aqueous solubility, suboptimal pharmacokinetics, low target site accumulation, high off-target localization, low efficacy and high toxicity. In order to overcome some of the barriers faced by low-molecular-weight chemotherapeutic drugs, various different nanomedicine formulations have been developed over the years, including e.g. liposomes, polymer-drug conjugates, polymeric micelles, polymeric and lipid nanoparticles, dendrimers and antibody-drug conjugates. Nanomedicines are carrier materials with a size of 1-100(0) nm and they are designed to improve the pharmacokinetic and biodistribution profile of encapsulated or conjugated (chemo-) therapeutics drugs, via improving aqueous solubility, avoiding accumulation in healthy tissues, improving target site localization, and increasing therapeutic index. These features have contributed to the approval and clinical use of approximately a dozen anticancer nanotherapeutics for the treatment of solid and hematological malignancies. These approved products include a variety of nanomedicine subtypes, such as non-PEGylated liposomal doxorubicin (Myocet^®^), PEGylated liposomal doxorubicin (Caelyx^®^, Doxil^®^), PEGylated liposomal irinotecan (Onivyde^®^), nanoparticle albumin-bound paclitaxel (Abraxane^®^), polymeric micelle-paclitaxel nanoformulation (Genexol^®^), and antibody-drug conjugates (Kadcyla^®^, Adcetris^®^, Mylotarg^®^, Besponsa^®^) 1. At clinical level, nanotherapeutics typically mostly reduce side effects, improving patient tolerability. For instance, administering Doxil^®^ results in less cardiotoxicity, neutropenia and alopecia as compared to free doxorubicin 2, 3. Analogously, Abraxane^®^ facilitates the use of a highly hydrophobic and highly potent taxane drug. As compared to the clinically routinely used Cremophor-based Taxol formulation, nanoparticle-based Abraxane^®^ can be given at a higher dose, in a shorter period of time, and without the need of co-medication 4.

Thus far, cancer nanomedicine formulations have largely failed to improve therapeutic outcomes in the clinic and in terms of patient survival. In 2016, Bind Therapeutics, a pharmaceutical company developing actively targeted anticancer nanotherapy (BIND-014), filed for bankruptcy, as it did not meet its endpoints in clinical trials. Similarly, several other promising cancer nanomedicine platforms based on polymeric nanoparticle (CRLX101) and polymeric micelle (NK105) have also reported disappointing clinical outcomes 5. These disappointing outcomes are considered to be due to the high inter- and intra-individual heterogeneity in the EPR (i.e. Enhanced Permeability and Retention) effect, which was initially presumed to be homogeneous and omnipresent in all tumor types. EPR is a pathophysiological phenomenon via which nanomedicine formulations are assumed to accumulate in cancerous tissues, and is based on leaky vasculature and impaired lymphatics 6. In animal models and particularly in human tumor, there are vast differences in EPR-contributing parameters, such as vessel density, perfusion and permeability, tumor stroma composition, interstitial fluid pressure, and lymphatic vessel density and functionality 7-9. These high levels of heterogeneity in key pathophysiological parameters contributing to EPR-mediated drug delivery are considered to be responsible for the sub-optimal therapeutic performances that have thus been achieved with cancer nanomedicines in the clinic 9, 10.

In order to boost the response rates of cancer nanomedicines in patients in the clinic, probes and protocols are needed that can address this high heterogeneity in EPR-mediated tumor accumulation. Various strategies, including pharmacological and physical priming treatments, have been used to modulate the tumor vasculature and microenvironment for improved EPR-mediated delivery of drugs and drug delivery systems. In the context of pharmacological interventions, strategies employing vascular normalization, vessel promotion, vascular disruption and vascular permeabilization have been employed to enhance EPR-mediated drug delivery to tumor. Physical modalities to modulate the tumor vasculature and microenvironment for improved drug delivery have encompassed radiotherapy, ultrasound, hyperthermia and photodynamic therapy 11-13.

In line with the above-mentioned notions, companion diagnostic and theranostic strategies to image EPR-mediated drug delivery are considered to be crucial for addressing EPR heterogeneity and improving anticancer nanotherapy in patients. For instance, imaging tools that can visualize different EPR characteristics in solid tumor and metastases can be highly useful in guiding clinical decision making, via the stratification of patients that are most likely to respond to nanotherapy due to prominent EPR, while excluding those subpopulations from nanotherapy regimes that are unlikely to respond due to poor baseline levels of EPR 14, 15. To this end, various theranostic strategies have been explored over the years. For instance, traditional non-invasive imaging modalities such as contrast-enhanced functional ultrasound (ceUS) 16, 17 and dynamic contrast-enhanced magnetic resonance imaging (DCE-MRI) 18 have been used to predict cancer nanomedicine accumulation at pathological sites. Other strategies have entailed the use of nanomaterial-based imaging agents to serve as companion diagnostics 19-22, and the co-encapsulation of imaging and therapeutic agents into a single nanomaterial (i.e. nanotheranostics) 14, 23-26. These strategies, which employ imaging in close conjunction with nanotherapy, have been shown to hold potential for patient stratification, via the identification of subpopulations most likely to benefit from nanotherapy. Additionally, these imaging approaches, when integrated in nanomedicine research at the pre-clinical level, can be used to better understand the fundamentals of the drug delivery process, for instance via the non-invasive visualization and quantification of biodistribution, target site accumulation and drug release 27, 28. We here summarize and discuss key concepts in imaging-assisted anticancer nanotherapy, and highlight companion diagnostic and theranostic strategies that can help to address the heterogeneity in EPR-mediated nanomedicine delivery.

## 2. Biodistribution imaging

**Figure 1:**
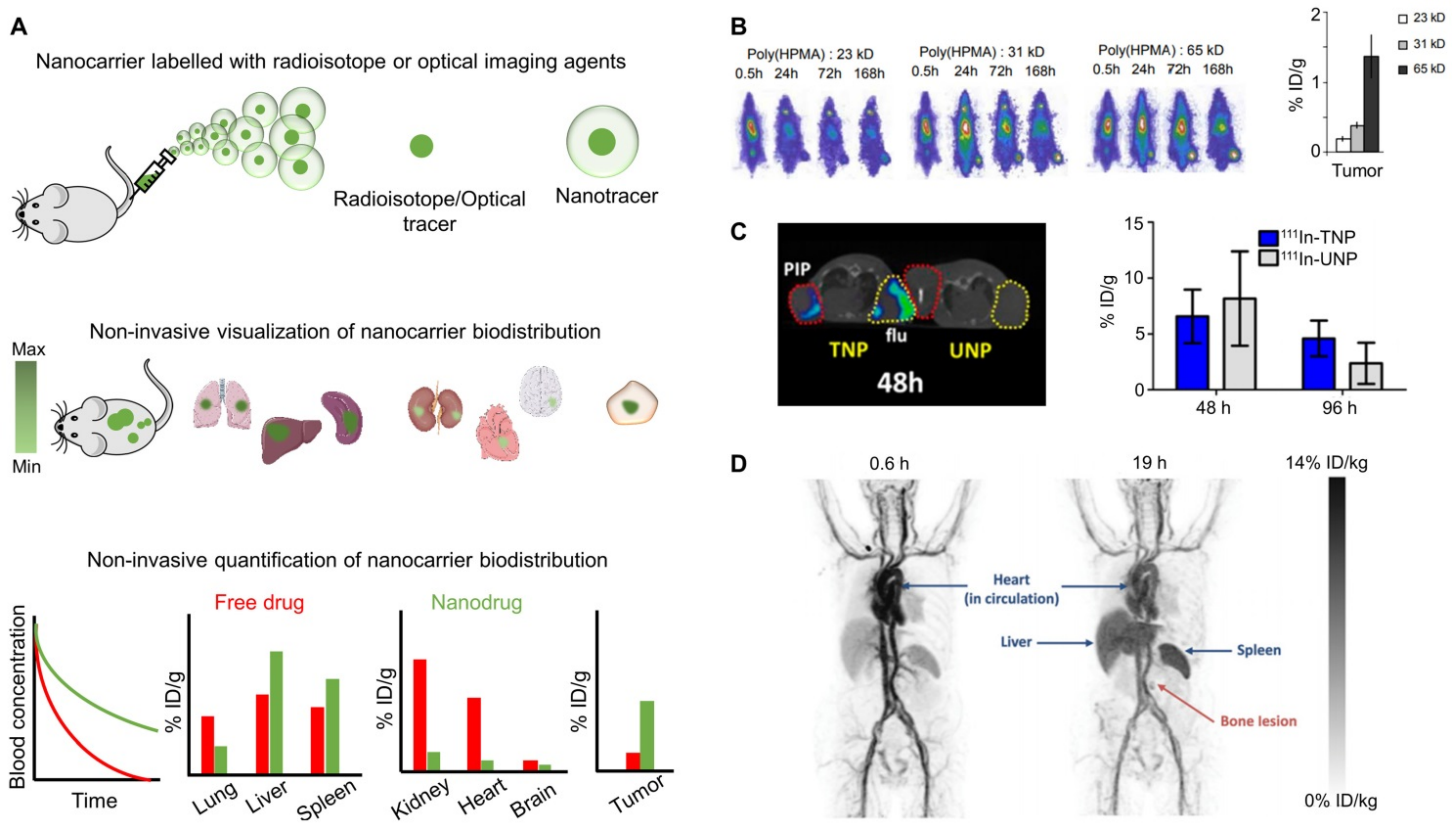
** Biodistribution imaging. (A)** Nanomedicines labeled with radioisotopes or optical imaging agents can be used to non-invasively visualize and quantify their pharmacokinetic properties and biodistribution, enabling systematic studies on nanomedicine-based drug delivery to pathological sites. **(B)** Prototypic poly(HPMA)-based drug carriers of varying molecular weights were radiolabeled with ^131^I, showing that higher molecular weight polymers circulate for longer periods of time and accumulate better in subcutaneous Dunning AT1 prostate carcinoma tumors implanted in the lower right leg of rats. **(C)** Qualitative SPECT/CT image in xenograft bearing mice showing that at 48 h p.i., ^111^In labeled PSMA targeted nanoparticle (TNP) accumulate in both PC-3 PIP, i.e. PSMA-positive (red circles) and PC-3 flu, i.e. PSMA-negative (yellow circles) tumors, while relatively low accumulation is observed for untargeted nanoparticle (UNP). Quantitative analysis shows better tumor retention of TNP than UNP at later time points, i.e. at 96 h p.i. **(D)** PET scans of a metastatic breast cancer patient showing that i.v. administered ^64^Cu-labeled HER2-targeted liposomes circulate in the bloodstream and eventually accumulate in the RES organs liver and spleen, as well as in a metastatic bone lesion. Images reproduced, with permission, from 23,30,31.

In order to visualize and quantify the circulation behavior, biodistribution and target site accumulation of cancer nanomedicines, imaging agents have been entrapped within or conjugated to nanoparticles, allowing them to be tracked via non-invasive imaging techniques 29-31. In this regard, well-established imaging techniques such as optical imaging (OI) and radionuclide imaging as well as a newer class of recently employed non-invasive imaging techniques have been used to monitor nanomedicine biodistribution and tumor accumulation (Figure 1A).

### 2.1 Optical imaging

In the context of OI, we have established hybrid CT-FMT (computed tomography-fluorescence molecular tomography) to enable CT-based assessment of mouse and organ anatomy together with FMT-based quantification of near-infrared fluorophore (NIRF) labeled polymeric nanocarriers based on poly(N-(2 hydroxypropyl) methacrylamide) (pHPMA). Furthermore, in order to facilitate FMT-based quantification of NIRF non-invasively, we performed scattering and absorption reconstruction to consider the different optical properties of organs, e.g. the high light absorption in highly perfused organs such as heart, liver, kidney as blood is the main near-infrared absorber *in-vivo* 32-34. This technique has proven to be very useful in preclinical research to assess nanomedicine biodistribution and EPR-based accumulation 6, 35. As an example, upon i.v. administration of NIRF-labeled pHPMA, we observed high levels of accumulation in organs of the reticuloendothelial system (RES), such as liver and spleen, while localization to kidney and bladder was low (as anticipated for nanocarriers). Additionally, the polymeric nanocarriers showed increasing levels of tumor accumulation over time, with concentrations peaking at 24 h post injection (p.i.) 29. Another study employed CT-FMT imaging to understand the intratumoral accumulation of differently sized-liposomes in two mammary adenocarcinoma models. Micro-CT in conjunction with iodinated liposomal contrast agent enabled spatiotemporal progression of tumor vasculature and perfusion CT allowed for quantitative assessment of tumor perfusion. FMT was employed to simultaneously visualize and quantify tumor accumulation of different size liposomes (labeled with different NIRF) in the same tumor. CT-FMT indicated that larger size liposomes (100 nm) accumulated much more in highly perfused tumor regions than in poorly perfused areas, which is in line with the principles of convective transport of larger particles. Additionally, active targeting of 100 nm liposome did not show any benefit in tumor deposition neither in highly perfused nor in poorly perfused areas. On the other hand, for the smaller size 30 nm liposomes, only active targeting enhanced the intratumoral deposition in poorly perfused tumor regions. This can be attributed to the relatively higher diffusivity of these smaller size targeted liposome into the tumor interstitium and the enhanced retention due to active targeting which further prevents the washout of these liposomes from the tumor interstitium back into the systemic circulation 36.

A key advantage of such OI techniques is that they allow for multispectral imaging, enabling head-to-head comparison of passively vs. actively targeted nanocarriers in the same tumor in the same mouse. As an example of this, it was shown in multiple mouse models that RGD-modified actively targeted pHPMA polymers achieve higher tumor concentrations at earlier time points as compared to non-modified passively targeted polymers, but that the overall levels of tumor accumulation (AUC) are significantly higher for non-modified polymers (as a result of longer circulation times) 37. Along the same line of thought, Tsvetkova et al studied the impact of actively riboflavin (RF)-targeted and passively targeted polymers, varying in sizes, on tumor accumulation and cellular internalization in different tumor compartments. The authors hypothesized that the size of an IgG antibody (12-15 nm) is ideal for a molecule to circulate long enough, take high benefit from passive accumulation in tumors and inflammatory sites, and still be small enough to penetrate deep into tissues and find the target. CT-FMT showed that active targeting with RF improves the tumor accumulation of 7 nm polymer, however, tumor accumulation of larger size polymers (13 nm) did not benefit from RF targeting. However, the total accumulated amount of larger polymers, targeted or not, was substantially higher than for the smaller ones. Apart from non-invasive OI techniques, the authors also employed invasive microscopy analyses to study nanoparticle distribution in different tumor cell compartments. These in-depth microscopy analyses highlighted the benefit of RF targeting in cellular internalization of both polymer sizes, proving that also the larger ones had reached the target sites. Interestingly, after performing immunohistochemical stainings for characterizing polymer uptake in different tumor cell compartments, it was found that RF-targeted 7 nm polymer was preferentially taken up by cancer cells, while the RF-targeted 13 nm polymer (being larger in size may slowly diffuse through the tumor extravascular space) showed preferential uptake by tumor-associated macrophages 38. This study demonstrates the usefulness of multiscale optical imaging, i.e. from CT-FMT to microscopy, in systematically investigating the added value of active targeting.

### 2.2 Radionuclide imaging

Besides OI, which is very practical preclinically, radionuclide imaging of nanomedicines has been extensively employed to assess pharmacokinetics, biodistribution and target site accumulation, both in animal models and in patients. As an example, iodine-131 (^131^I)-radiolabeled pHPMA polymers of varying molecular weights, nature and functional groups were employed to study the influence of size, charge and side group modification on their tumor targeting ability in rats bearing subcutaneous Dunning AT1 prostate carcinoma tumors. The authors demonstrated that larger polymers (10-20 nm) circulate longer than smaller polymers (2-3 nm and 4-6 nm), as a result of less efficient renal excretion, and that this prolonged presence in the bloodstream contributes to more efficient EPR-based tumor targeting (Figure 1B). Upon functionalization pHPMA with carboxyl or hydrazide group, with drugs and drug linkers, as well as with peptides, the tumor accumulation of the polymers decreased, as a result of reduced circulation times and stronger kidney accumulation 30. To further improve intra-tumoral deposition, injection of 31 kDa and 65 kDa radiolabeled pHPMA was preceded by radiotherapy and hyperthermia in three different tumor models. Gamma-scintigraphy revealed that pre-treating tumors with radiotherapy could enhance the tumor accumulation of pHPMA in all tumor models, while the effect of hyperthermia was shown to be dependent on the tumor model. These effects were further confirmed using a chemically modified, i.e. drug-loaded, pHPMA, which showed enhanced tumor accumulation only after pre-treating the tumors with radiotherapy 39.

Radionuclide imaging has also been employed to investigate the tumor accumulation of passively and actively targeted nanocarriers. For instance, actively prostate-specific membrane antigen (PSMA) -targeted nanoparticles and their corresponding passively targeted controls were radiolabeled with indium-111 (^111^In) to compare their tumor accumulation in mice containing PSMA-positive PC3 PIP tumors. As illustrated in Figure 1C, *in vivo* SPECT imaging showed an accumulation of ~6% ID/g for ^111^In-labeled actively targeted nanoparticle at 48 h p.i. and levels remained relatively constant over time. Conversely, ^111^In-labeled untargeted nanoparticle showed a higher tumor uptake of ~8% ID/g at 48 h p.i., followed by a more prominent clearance between 48 and 96 h 31. Such image-guided analyses are very helpful to better understand the principles of actively targeted drug delivery.

Also at the clinical level, the biodistribution and tumor accumulation of cancer nanomedicines have been studied upon radiolabeling 40. In one of the first pioneering studies, in this regard, Harrington and colleagues radiolabeled PEGylated liposomes with ^111^In to evaluate their pharmacokinetics, biodistribution and tumor accumulation in 17 patients with locally advanced cancers. Gamma camera imaging demonstrated clear persistence in the cardiac pool signal even after 72 h p.i., revealing that i.v. administered ^111^In-labeled PEGylated liposomes circulate for long periods of time. The liposomes showed significant accumulation in liver, spleen and bone marrow, which are known to be the major RES organs and involved in the clearance of nanoformulations from the bloodstream. Liposome accumulation could be observed in 15 out of 17 tumor lesions, with the highest uptake levels in head and neck cancer, intermediate in lung cancer and lowest in breast cancer 41. In another study, Koukourakis and colleagues employed SPECT and scintigraphic imaging for monitoring the biodistribution of a technetium-99m (^99m^Tc)-radiolabeled Caelyx^®^, which was administered in combination with radiotherapy to patients with sarcoma, non-small cell lung cancer (NSCLC) and head and neck (HNC) cancer. Caelyx^®^ was found to accumulate significantly more in all tumors when compared to the surrounding healthy tissue in case of sarcoma, or when compared to cardiac blood pool for NSCLC and HNC patients 42, 43.

In a similar setup, but with a more advanced imaging hardware (hybrid PET/CT), Merrimack Pharmaceuticals studied the biodistribution of ^64^Cu-labeled HER2-targeted PEGylated liposomal doxorubicin (MM-302) in patients with HER2 positive metastatic breast cancer. As shown in Figure 1D, PET/CT analysis exemplified long circulation properties of ^64^Cu-MM-302, alongside low levels of localization in most healthy tissues (e.g. muscle, lung, bone marrow) and strong accumulation in RES organs. With regard to tumor accumulation, significant inter- and intra-individual variation was observed in primary breast carcinomas as well as in different metastatic lesions (in lymph node, lung, liver, bone and brain). These translational efforts are very valuable for furthering clinical progress in cancer nanomedicine, particularly by enabling physicians to obtain non-invasive imaging biomarkers for patient stratification (see below, chapter 3) 23.

### 2.3 Emerging imaging techniques

Apart from the above-mentioned established modalities, strategies based on magnetic particle imaging (MPI), photoacoustic imaging (PAI) and multispectral optoacoustic tomography (MSOT) are emerging as new technologies for non-invasive imaging. In many of these new modalities, nanoparticles are used as contrast agents. Thus far, however, only few studies have employed these techniques to systematically analyze nanoparticle biodistribution and target site accumulation. It is anticipated that will be increasingly done in the future, employing MPI to study the biodistribution of superparamagnetic iron oxide nanoparticles, and PAI and MSOT to evaluate the *in vivo* fate of gold- and carbon-based nanoparticles, as well as e.g. hemoglobin-, melanin- and porphyrin-containing nanomaterials 44-46.

## 3. Drug release imaging

**Figure 2:**
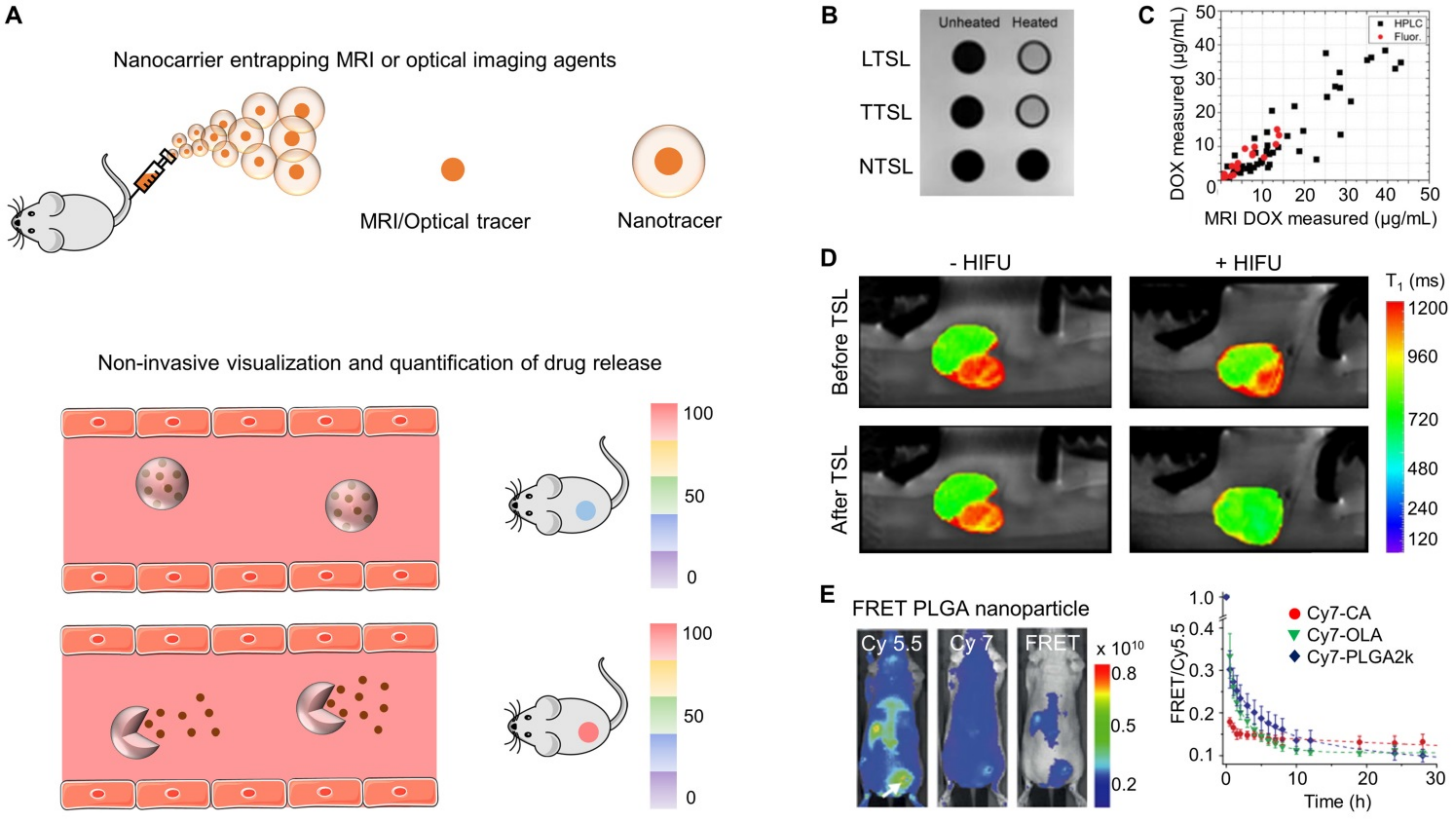
** Drug release imaging. (A)** Nanoformulations entrapping MRI or OI agents can generate different signals when the contrast agent is in entrapped vs. in free form, allowing for non-invasive monitoring of drug release. **(B)** Liposomes loaded with ProHance^®^, an MRI contrast agent, show low MRI contrast while being intact at physiological temperature. Upon hyperthermia, low and traditional temperature-sensitive liposomes (LTSL and TTSL) re-order their lipid biolayer, resulting in ProHance^®^ release and in an increase in MRI signal. This does not occur for non-temperature-sensitive liposomes (NTSL). **(C)** MRI signal enhancement upon hyperthermia treatment of temperature-sensitive liposomes (TSL) shows a good correlation with doxorubicin release, exemplifying the usefulness of such setups for non-invasive release monitoring. **(D)** MRI scan of 9L gliosarcoma rat tumors demonstrating MRI contrast agent (and drug) release from temperature-sensitive liposomes (TSL) upon focused ultrasound-based hyperthermia treatment. **(E)** PLGA nanoparticles co-loaded with Cy-5.5 as a FRET donor and Cy-7 as FRET acceptor enable the visualization and quantification of nanocarrier-drug association and dissociation (i.e. drug release) in MDA-MB-231 breast cancer xenograft tumors. Images reproduced, with permission, from 48,49,53,57.

Apart from achieving an optimal biodistribution and target site accumulation, it is also of crucial importance that the therapeutic payload is released from nanocarriers at the target site. To non-invasively visualize and quantify drug release, cancer nanotheranostics have been developed in which imaging agents and chemotherapeutic drugs are co-entrapped into the same delivery system. In such setups, the imaging agent must render a different signal when it is bound/entrapped in the nanomaterial versus when it is present in unbound/free form upon release. For this reasons, PET and SPECT-based probes are less suited for drug release imaging. Instead, optical agents and MRI-based contrast media possess features suitable for this purpose 27, 47 (Figure 2A).

### 3.1 Magnetic resonance imaging

MRI-based approaches have been widely employed for monitoring drug release. Since the imaging signal from MR contrast media depends on the interaction with bulk water, entrapped paramagnetic MR agent in a water-impermeable nanocarrier only induces a slight T_1_ shortening, whereas its release into an aqueous media results in hyperintense signals in T_1_-weighted MRI, thereby serving as a reporter for monitoring drug release. As an example, De Smet et al. designed NTSL (non-temperature sensitive liposomes) and TSL (temperature sensitive liposomes), both co-loaded with doxorubicin and the MR contrast agent ProHance (i.e., [Gd(HPDO3A)(H_2_O)]), for monitoring drug release via MRI. At physiological temperature (T<T_m_), intact NTSL and TSL showed hardly any MRI contrast; upon increasing the temperature (T>T_m_), the lipid bilayer of the TSL formulation re-arranged, releasing ProHance and generating a significantly increased MRI signal (Figure 2B) 48. In this and similar preclinical setups, a good correlation has been demonstrated between contrast agent release from TSL and doxorubicin release (Figure 2C) 49.

MRI offers high spatial resolution allowing the study of intra-tumoral drug distribution upon triggered drug release from nanocarriers. For instance, thermosensitive liposomes co-loaded with doxorubicin and Gd-DTPA have been utilized to non-invasively assess intra-tumoral distribution of the released cargo in tumor bearing mice using MRI. In particular, after hyperthermia treatment, the MRI enhancement from the released Gd-DTPA was particularly high in the tumor periphery, while minimal signal was observed in the tumor core. The observed differences in drug distribution can be attributed to the heterogeneity in intra-tumoral vascularization. This pattern correlated well with the intra-tumoral distribution of released doxorubicin (analyzed via fluorescence microscopy), illustrating the potential of MRI-based techniques for non-invasively monitoring both the temporal and the spatial aspects of (triggered) drug release from nanocarriers in tumors 50.

The integration of MRI in ultrasound-mediated hyperthermia protocols is beneficial for optimizing triggered drug release. Applying FUS (i.e. focused ultrasound) at a target lesion, for instance a tumor, causes local heating thereby facilitating triggered drug release from TSL. This is generally performed under MRI guidance, which provides anatomical information for the delineation of the targeted lesion and also provides real-time feedback on the temperature increase (via MR thermometry). Such MRI-guided FUS setups have been used to ensure local heating of only pathological sites, thereby facilitating triggered drug release from TSL with high spatial specificity 51, 52. As an example, Grüll and colleagues employed a clinical MR-FUS setup in rats bearing 9L gliosarcoma tumors to optimize the release of doxorubicin (via monitoring ProHance release) from TSL upon mild hyperthermia. MRI enabled anatomical localization of the tumor as well as real-time temperature mapping, thereby triggering ProHance release from liposomes only at the tumor site, and not in surrounding muscle tissue (Figure 2D). A good correlation was observed between the relaxivity enhancement caused by the released ProHance and the concentration of free doxorubicin in the tumor, exemplifying the value of using MRI to guide FUS-mediated local drug delivery and release 53.

An additional application/advantage of using imaging to tailor locally triggered drug release is that the time point of triggering can be optimized, i.e. applying the stimulus (e.g. hyperthermia) at those time points at which nanocarrier accumulation at the target site is maximal. This can e.g. be achieved by co-entrapping two different MRI contrast agents in a single nanocarrier. For example, Onuki et al co-entrapped iron oxide nanoparticles, Gd-DTPA and 5-fluorouracil (5-FU) into PLGA nanoparticles and employed multiplexed MRI to track tumor accumulation and drug release. Upon i.v. administration, the entrapped iron oxide nanoparticles were used to assess tumor accumulation via T_2_-weighted imaging, and release of Gd-DTPA - which was used as a surrogate marker to visualize the release of 5-FU from the nanoparticles - was monitored via T_1_-weighted imaging 54.

### 3.2 Optical imaging

Apart from MRI, optical imaging strategies based on quenching/de-quenching and FRET (fluorescence resonance energy transfer) have also been implemented for monitoring drug release 47, 55, 56. In a proof-of-concept study by Mulder and colleagues, Cy5.5-conjugated PLGA nanocarrier material serving as FRET donor and encapsulated Cy7-X model drugs (i.e. a NIRF dye with different lipid tail components: referred to here as X) serving as a FRET acceptor were used to study the nanocarrier-drug association and dissociation (release) kinetics in real-time using *in vivo* optical imaging in mice bearing MDA-MB-231 breast cancer xenografts. Upon i.v. injection, the authors determined the FRET/Cy5.5 intensity ratio to assess the nanocarrier-drug association, observing that model drugs with higher hydrophobicity and higher miscibility with the polymeric matrix had the strongest nanocarrier association and hence prevented premature drug release (Figure 2E). This notion was confirmed by evaluating the *in vivo* behavior of distinct nanocarrier-doxorubicin prodrugs (similar lipid tail compositions as employed in Cy7-X) in mice with 4T1 tumors. As hypothesized, the group with the strongest nanocarrier-doxorubicin association exhibited better anti-tumor efficacy as compared to other treatment groups 57.

Together, these studies exemplify the usefulness of MR and OI agents for real-time monitoring and guidance of triggered drug release and nanocarrier-drug association and dissociation *in vivo*. Such tools are valuable for optimizing treatment protocols and for systematically studying nanomedicine stability, drug release and drug efficacy.

## 4. Patient stratification

**Figure 3:**
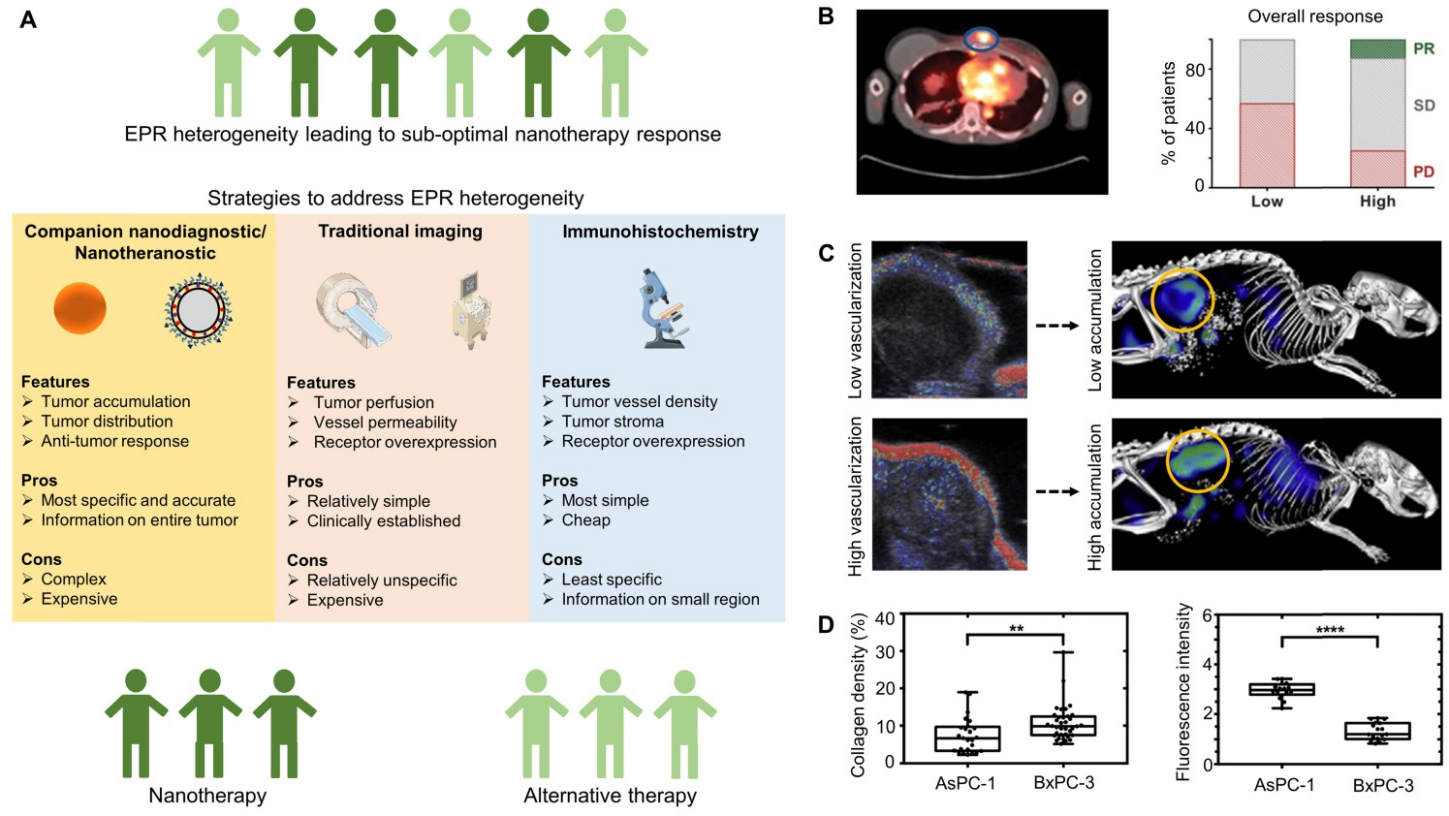
** Patient stratification. (A)** Strategies to address EPR heterogeneity by identifying patients that are most amenable to nanotherapy response. **(B)** Left panel: PET-CT scan of a metastatic breast cancer patient showing that a nanotheranostic formulation, i.e. ^64^Cu-labeled HER2-targeted doxorubicin liposomes (MM302), can be used to monitor accumulation in a metastatic sternal mass (blue circle). Right panel: Correlation between accumulation of MM-302 and overall response, showing that higher uptake results in better overall response rates as compared to lower uptake. PR: partial response. SD: stable disease. PD: progressive disease. **(C)** Contrast-enhanced ultrasound (CEUS) imaging of CT26 murine colon carcinoma tumors showing that the level of functional tumor vascularization correlates polymeric nanocarrier accumulation in tumors (yellow circles). **(D)** Immunohistochemical analysis of collagen in two different pancreatic cancer models exemplifies that tumors with a relatively high collagen density accumulate less fluorescently-labeled model drug. Images reproduced, with permission, from 16,23,66.

Due to high inter- and intra-individual heterogeneity in EPR-mediated nanocarrier accumulation in tumors and metastases, cancer nanomedicines have not been performing optimally in patients 10, 15 Imaging this heterogeneity in EPR, either directly using nanotheranostics or indirectly via visualizing key processes such as tumor blood vessel perfusion or receptor over-expression (in case of actively targeted nanomedicines) may help to improve nanomedicine performance 9. Such strategies are considered highly useful for facilitating clinical decision-making, e.g. with regard to the preselection of patients most likely to respond to nanotherapy (because of prominent EPR) and for excluding patients who are unlikely to respond (because of low EPR) 58. As illustrated in Figure 3A, several imaging-based strategies can be employed to visualize and quantify heterogeneity in EPR-based nanomedicine accumulation in tumors.

### 4.1 Companion nanodiagnostics and nanotheranostics

Companion nanodiagnostics and nanotheranostics have been employed both in preclinical and clinical setups, and they allow for the most accurate prediction/assessment of nanomedicine localization at pathological sites. At preclinical level, drug-free nanoreporter liposomes labeled with zirconium-89 (^89^Zr) have been shown to be useful as a companion diagnostic to predict Doxil^®^ accumulation and efficacy in a breast cancer mouse model 20. Similarly, other PET radionuclides such as copper-64 (^64^Cu) have been used to label drug-free liposomes in order to predict the accumulation and anti-tumor response of similar sized nanodrugs 59. A second elegant example of a companion nanodiagnostic approach relies on the repurposing of ferumoxytol, which is a ~30 nm-sized iron oxide nanoparticle formulation that is clinically approved for the treatment of anemia, and that can be used off-label as an MRI contrast agent to visualize and quantify nanoparticle accumulation in tumors. Despite differences in size and physicochemical properties, a good correlation was observed between the tumor concentrations of ferumoxytol and that of ~100 nm-sized polymeric nanoparticles loaded with paclitaxel 60. Moreover, those tumors accumulating ferumoxytol most efficiently displayed the highest levels of anti-tumor efficacy upon treatment with paclitaxel-containing polymeric nanoparticles. These preclinical proof-of-concept studies exemplify the potential of using companion nanodiagnostics to predict the accumulation and efficacy of nanotherapeutics.

Extending these efforts towards the clinic, scientists at Merrimack Pharmaceuticals have recently reported two trials in which companion nanodiagnostics and nanotheranostics are employed. In the case of former, as in the example described above, ferumoxytol was used in conjunction with MRI to demonstrate that patients with tumors that accumulate ferumoxytol respond better to treatment with Onivyde^®^ than patients with low levels of ferumoxytol tumor accumulation 61. One of the key advantages of such strategies involving the use of clinically already approved nanoparticles as companion nanodiagnostics is that they, in principle, allow for immediate clinical implementation. An important disadvantage is that there unavoidably are differences in physicochemical characteristics between companion nanodiagnostics and nanotherapeutics, resulting in differences in pharmacokinetic behavior, biodistribution and target site accumulation. Whether the accuracy of the imaging information obtained is accurate enough to properly predict nanotherapy accumulation and efficacy in prospective clinical trials needs to be assessed in the near future.

Nanotheranostic formulations, which contain both drugs and imaging agents, can be employed to avoid the above issue. Nanotheranostics obviously provide the most accurate information on target site accumulation, but as compared to companion nanodiagnostics, they are arguably more difficult to translate to and implement in the clinic. In a pioneering trial, published in the same volume of Clinical Cancer Research, scientists from Merrimack Pharmaceuticals labeled MM-302 (HER2-targeted liposomal doxorubicin) with copper-64 (^64^Cu) and employed hybrid PET/CT to directly visualize tumor accumulation and therapeutic response in HER2-positive metastatic breast cancer patients. Despite the high intra- and inter-individual variability in the accumulation of ^64^Cu-MM 302 in different metastatic lesions, it was found that higher levels of tumor localization corresponded to more favorable treatment outcomes, while tumors with low levels of accumulations generally did not respond well 23 (Figure 3B). Theranostic nanomedicine formulations thus allow for the direct visualization of tumor accumulation, and they consequently seem to be very useful in clinical trials to stratify patients with a higher likelihood to respond to nanotherapeutic interventions.

Apart from predicting the efficacy of nanotherapy interventions, nanotheranostics may also be very useful in guiding tumor priming treatments. As an example for this, Merrimack scientists used cyclophosphamide (which is known to reduce tumor cell density, interstitial fluid pressure and improve perfusion) to enhance the delivery of ^64^Cu-labeled MM-302 in different tumor models. It was found that the timing and dosing of cyclophosphamide treatment were crucial for priming of the tumor microenvironment and for achieving optimal deposition of ^64^Cu-labeled MM-302 in tumors (as measured by PET-CT) 62, 63. Similarly, such theranostic setups can help to advance tumor priming via vascular normalization, for which the beneficial effects on drug delivery also highly depend on the timing and dosing of anti-angiogenic therapies 64, 65. In the clinic, visualizing and quantifying the tumor accumulation and distribution of theranostic nanomedicines may guide oncologists in optimizing the timing and dosing of priming treatments, to thereby achieve optimal drug delivery and therapeutic outcomes.

Despite the above-mentioned potential of nanotheranostics in optimizing treatment regimens and stratifying patients, a downside of using nanotheranostic constructs and concepts is that specialized equipment (for radiolabeling and PET-CT imaging) is needed at the clinical centers in which they are being tested/employed, and also that additional regulatory documentation and evaluation is required to enable their use in patients.

### 4.2 Conventional imaging techniques

Besides using companion nanodiagnostics and nanotheranostics, a more simple and straightforward (but likely less specific) strategy for patient stratification would be to employ traditional non-invasive imaging modalities such as contrast-enhanced MRI or US. These techniques can provide useful information on EPR-relevant biomarkers, such as relative blood volume, vessel size, vessel perfusion and vessel permeability, and they may thereby indirectly predict nanotherapy accumulation and efficacy. As an example, it was shown that i.v. administered MRI contrast agents (Gd-DOTA and ultrasmall superparamagnetic iron oxide nanoparticles) enabled the characterization of the tumor microvascular structure (vessel size, blood volume fraction) and vessel permeability in eight different tumor models. Upon initial MRI assessment, tumor-bearing mice received i.v. injections of fluorescent nanoparticles and 2D-FRI-based optical imaging was used to assess nanoparticle accumulation in tumors. Despite the high inter-tumor heterogeneity observed in vascular characteristics and nanoparticle accumulation, it was found that a combinatorial assessment of vessel permeability and relative blood volume fraction can predict the (in-)efficiency of tumor accumulation quite accurately 18. Along the same line of thinking, we used contrast-enhanced ultrasound (CEUS) imaging to capture tumor perfusion in a murine colon carcinoma model, and showed that the degree of tumor vascularization as characterized by CEUS correlated well with the tumor accumulation of fluorophore-labeled polymeric nanocarriers 16 (Figure 3C). Another interesting study employed US elastography to measure shear modulus, i.e. tissue stiffness, and investigated the impact of shear modulus on drug delivery in two pancreatic cancer xenograft models. It was found that tumors with high collagen content had a higher shear modulus, and such high tissue stiffness led to collapsed vessels and consequently impeded drug delivery, while tumors with a lower shear modulus, i.e. low collagen content, showed higher drug uptake 66.

Recently, imaging-based simulation of the vascular network in tumors has also allowed for better understanding of the tumor microenvironment when predicting nanomedicine performance. To this end, Esposito et al employed computational modelling (based on data obtained from *ex vivo* imaging) and compared the mathematical simulations to *in vivo* tumor perfusion and uptake of a MRI contrast agent, i.e. Gd-DTPA, in two different tumor models. Despite heterogeneity in perfusion within the same tumors and across different sub-types, a fairly good association was observed between the model predictions of tumor perfusion and uptake, and those obtained using experimental *in-vivo* data. These results demonstrate the feasibility of computational modelling to predict drug uptake across different subjects and allows for the identification of subpopulations likely to benefit from treatment 67.

Beyond visualizing and quantifying the tumor accumulation of nanomedicines, we consider it crucial to also develop tools and technologies for imaging tumor penetration and intratumoral distribution. These parameters are as important as overall tumor accumulation with regard to predicting/ensuring good nanotherapy outcomes. In this regard, Sulheim et al. employed various imaging modalities to predict the accumulation and intratumoral distribution of nanoparticles in 5 different tumor models. In the context of tumor penetration, diffusion-weighted MRI, which assesses the diffusivity of water molecules, did not correlate well with the nanoparticle uptake 68. The nanoparticles used in the study, however, were rather large (100 nm), and it seems reasonable that diffusion-weighted MRI is more suitable for predicting the tumor accumulation, penetration and/or distribution of substantially smaller carrier materials, such as linear polymers and antibodies, as well as that of (released) drug molecules. Given the increasingly recognized importance of tumor penetration and intratumoral distribution, dedicated efforts are needed to develop probes and protocols to visualize and quantify these parameters, in mouse models and in patients. This would be helpful not only in the context of nanomedicine and drug delivery, but also when exploring novel tumor priming treatments and combination therapies (including immunotherapy).

### 4.3 Immunohistochemistry

Classical immunohistochemistry may also hold value for “imaging-based” prediction of nanomedicine accumulation and performance. It can be employed for the assessment of nanomedicine-relevant biopsy biomarkers, such as vessel density, macrophage density, collagen density and target receptor expression, and it may as such be able to indirectly predict nanotherapy accumulation and efficacy. As an example, in the context of visualizing receptor overexpression, prior to administering Herceptin^®^ (trastuzumab; a monoclonal antibody therapeutic that binds to HER2 and blocks HER2 signaling) for the treatment of metastatic breast cancer, patient biopsies are first examined by immunohistochemistry to assess the levels of HER2. Subsequently, patients with sufficiently high levels of HER2 expression are then treated with Herceptin^®^, while patients that have no or low HER2 expression are excluded from treatment, resulting in efficient and personalized targeted treatment.

Analogously, in the context of visualizing tumor blood vessel density, a clinical study performed in the 1990's employed CD31 staining to assess microvascular density in the biopsies of nine patients with non-small cell lung cancer. These patients subsequently received radiolabeled Doxil^®^/Caelyx^®^ (PEGylated liposomal doxorubicin), enabling tracking via SPECT-based gamma-scintigraphy. This study demonstrated that tumor microvascular density correlates well with the level of Doxil^®^/Caelyx^®^ accumulation, and also that patients with higher levels of accumulation responded better than those with low accumulation 42. Despite this initial proof-of-concept provided in late 1990's, no follow-up efforts have been documented thus far. In a related study, it was demonstrated that the density of collagen, as analyzed via Masson trichrome staining, varied greatly in different pancreatic tumor models, and that tumors with high collagen density showed a poor drug penetration 66 (Figure 3D). Along the same line of thinking, it may be worthwhile to consider staining and quantifying (specific subsets of) tumor-associated macrophages in biopsies, as it is increasingly recognized that macrophages are responsible for retaining nanomedicines in tumors 60.

In summary, the above-mentioned imaging strategies, which include companion nanodiagnostics, nanotheranostics, traditional non-invasive imaging techniques and immunohistochemistry have been showing promise in capturing the heterogeneity in EPR effect, and they can potentially be employed in the clinic to guide nanomedicine-based interventions, via the identification of patients with sufficiently high levels of EPR to ensure good therapeutic outcomes.

## 5. Conclusion

Imaging will play an important role in the future of cancer nanomedicine. By encapsulating contrast agents in drug-free or drug-containing nanomedicines, pharmacokinetic and biodistributional properties as well as nanomedicine accumulation and distribution at pathological sites can be assessed non-invasively and quantitatively. This will be important for patient stratification and for making the clinical translation of cancer nanomedicines more efficient. By incorporating MRI contrast media or optical imaging agents into nanocarriers, it is possible to non-invasively assess drug release *in vivo* in real-time. This will provide useful insights in drug release kinetics *in vivo* and, more importantly, will be highly valuable for optimizing/guiding the performance of triggerable nanomedicine formulations, such as Thermodox^®^. Furthermore, non-invasive and invasive (i.e. biopsies) imaging-based assessment of tumor blood vessel density, perfusion and permeability, as well as of the composition of the tumor ECM is considered to be important for achieving progress in the cancer nanomedicine field. In order to increase the clinical impact of cancer nanomedicines, rational strategies and realistic translational scenarios are needed to guide EPR-based tumor-targeted treatments. Imaging will play a crucial role in this and it is expected that several “nanotheranostic” concepts will soon start to contribute to more efficient nanomedicine-based treatments for patients suffering from cancer.
